# SMIM1, carrier of the Vel blood group, is a tail-anchored transmembrane protein and readily forms homodimers in a cell-free system

**DOI:** 10.1042/BSR20200318

**Published:** 2020-05-07

**Authors:** Anja Nylander, Pawel Leznicki, Karina Vidovic, Stephen High, Martin L. Olsson

**Affiliations:** 1 Department of Laboratory Medicine, Division of Hematology and Transfusion Medicine, Lund University, BMC C14, SE-22184 Lund, Sweden; 2 Department of Internal Medicine, Kristianstad-Hässleholm Hospitals, Kristianstad, Region Skåne, Sweden; 3 School of Biological Sciences, University of Manchester, Oxford Road, Manchester M13 9PT, U.K.; 4 Department of Clinical Immunology and Transfusion Medicine, LabMedicine, Office for Medical Services, Region Skåne, Sweden

**Keywords:** Cell-free translation, SMIM1, Tail-anchored protein, Transfusion medicine, Transmembrane protein, Vel blood group system

## Abstract

Antibodies to the Vel blood group antigen can cause adverse hemolytic reactions unless Vel-negative blood units are transfused. Since the genetic background of Vel-negativity was discovered in 2013, DNA-based typing of the 17-bp deletion causing the phenotype has facilitated identification of Vel-negative blood donors. *SMIM1*, the gene underlying Vel, encodes a 78-amino acid erythroid transmembrane protein of unknown function. The transmembrane orientation of SMIM1 has been debated since experimental data supported both the N- and C-termini being extracellular. Likewise, computational predictions of its orientation were divided and potential alternatives such as monotopic or dual-topology have been discussed but not investigated.

We used a cell-free system to explore the topology of SMIM1 when synthesized in the endoplasmic reticulum (ER). SMIM1 was tagged with an opsin-derived N-glycosylation reporter at either the N- or C-terminus and synthesized *in vitro* using rabbit reticulocyte lysate supplemented with canine pancreatic microsomes as a source of ER membrane. SMIM1 topology was then determined by assessing the N-glycosylation of its N- or C-terminal tags. Complementary experiments were carried out by expressing the same SMIM1 variants in HEK293T/17 cells and establishing their membrane orientation by immunoblotting and flow cytometry.

Our data consistently indicate that SMIM1 has its short C-terminus located extracellularly and that it most likely belongs to the tail-anchored class of membrane proteins with the bulk of the polypeptide located in the cytoplasm. Having established its membrane orientation in an independent model system, future work can now focus on functional aspects of SMIM1 as a potential regulator of erythropoiesis.

## Introduction

Antibodies directed toward the Vel blood group antigen were first described in 1952 as a result of hemolytic transfusion reactions [1]. The Vel-negative (Vel–) phenotype is comparatively rare, being approximately 1 in 1200 in Sweden but approximately 1 in 4000 in the United States and Europe, thus constituting a challenge in clinical transfusion medicine to find matched blood units for patients who make anti-Vel. Until 2013 the search for Vel– blood was totally dependent on scarce human antisera, but after we and others [2–4] identified the gene and underlying carrier for Vel, the availability of Vel– blood has increased thanks to genotyping and the production of monoclonal anti-Vel reagents [5,6].

**Figure 1:**
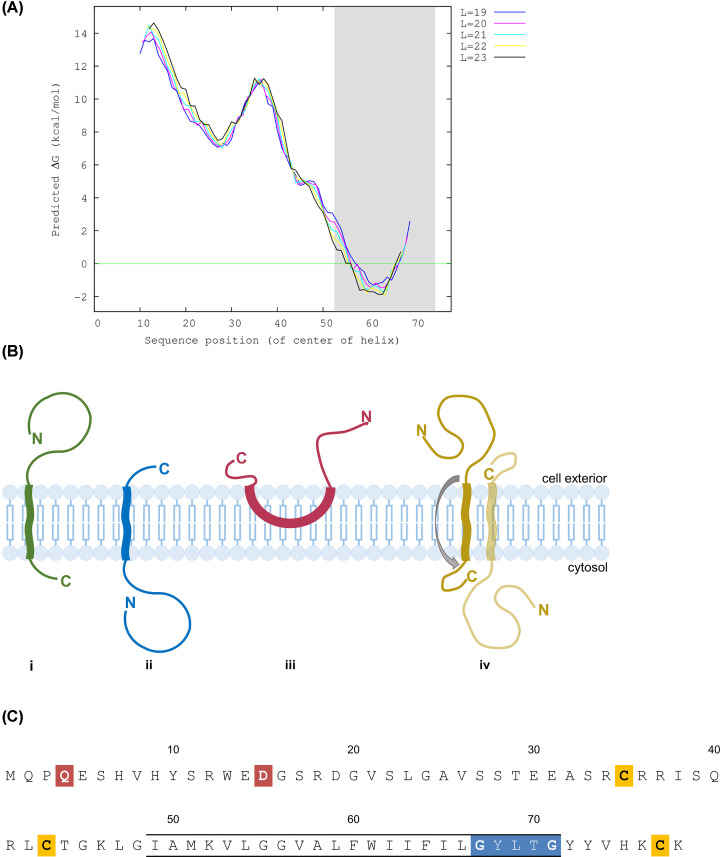
Predicted topology of SMIM1 based on free energy, sketches of transmembrane proteins and SMIM1 sequence The tool ‘prediction of ΔG for helix insertion prediction’ [8] (http://dgpred.cbr.su.se/index.php?p=fullscan) calculates the free energy associated with membrane insertion of SMIM1 into the phospholipid bilayer for transmembrane region lengths of 19–23 amino acids (**A**). Values approximately and under 0 correspond to ΔG in favor of membrane insertion. C-terminus display favorable membrane insertion. No hydrophobic N-terminal signal sequence is predicted, thus corresponding to SMIM1 having a tail-anchored orientation. (**B**) Cell membrane with sketches of: (**i**) type I (or III if no cleavable ER signal sequence), (**ii**) tail-anchored transmembrane protein as well as (**iii**) monotopic integral membrane protein and (**iv**) dual-topology protein. (**C**) SMIM1 amino acid sequence with predicted transmembrane region indicated with lines, cysteines marked in yellow, GxxxG motif marked in blue and amino acids mutated to insert N-glycosylation sites indicated in red.

Vel-negativity is caused by a 17-bp deletion (rs566629828) in the Small Integral Membrane Protein 1 (*SMIM1*) gene on chromosome 1. The *SMIM1* gene consists of four exons and encodes a 78-amino acid long protein with a transmembrane region residing close to the C-terminus. The topology of the membrane inserted protein has been enigmatic, with multiple computational algorithms predicting different results. Protein topology calculators rely on the distribution and proportion of hydrophobic amino acids to predict transmembrane protein regions, as well as machine learning. Depending on which algorithm was used, different orientations of the SMIM1 protein were predicted upon discovery of the molecule [7]. Another approach to suggest membrane orientation is the calculated free energy change associated with the entry of specific amino acid sequences into the lipid bilayer via the Sec61 translocon (Figure 1A) [8].

SMIM1 is a red blood cell (RBC) membrane protein of limited size and initial experiments indicated that the N-terminal portion could be cleaved with α-chymotrypsin from the surface of intact RBCs [2]. Thus, although it lacked a cleavable N-terminal endoplasmic reticulum (ER) signal sequence, it appeared otherwise similar to abundant RBC membrane proteins like the glycophorins. Although Glycophorin A is a type I transmembrane protein, guided to the ER membrane by an N-terminal cleavable signal sequence, Glycophorin C lacks such a signal sequence, but still assumes a transmembrane topology where its N-terminus is located outside the plasma membrane. This latter topology is sometimes classified as a type III transmembrane protein [9,10]. On this basis, our initial prediction was that SMIM1 may also be a type III protein, an orientation that would also allow for the major portion of the protein to be displayed on the outside of the cell thereby providing multiple immunogenic sites that might explain the diversity between anti-Vel antibodies from different individuals [11]. However, in 2015 Arnaud et al*.* [7] published data strongly suggesting that SMIM1 orients itself as a type II transmembrane protein with a short extracellular C-terminus of approximately five to ten amino acids, on which the Vel antigen is carried. This topology is reminiscent of a distinct group of integral membrane proteins known as tail-anchored proteins [9,10] (Figure 1B).

In order to further investigate the orientation of SMIM1, we turned to a cell-free translation and membrane insertion system independent from previous methods used to investigate the topology of SMIM1. The cell-free system would allow us to investigate the transmembrane topology in detail and detect not only whether the protein was either simply N-out or C-out, but also to address alternative possibilities [12]. Hence, we also considered that SMIM1 could be a monotopic integral membrane protein [12], in which both the N- and C-termini are exposed extracellularly (Figure 1B), or a dual topology protein with individual polypeptides having either their N- or C-terminus outside the cell [13] (Figure 1B). Crucially, once transmembrane topology has been established at the ER, the relative orientation of these proteins is maintained following their transport to the plasma membrane.

As was initially shown for Vel-reactive RBC membrane components [14], the apparent size of SMIM1 by Western blotting is dependent on the presence or absence of a reducing agent during sample processing. Thus, reducing conditions result in products of roughly half the size of those seen under non-reducing conditions [2]. We therefore speculated that disulfide bonds between one or more of the cysteines located at positions 35, 43 and 77 of SMIM1 may enable the formation of SMIM1 dimers (Figure 1C). However, the SMIM1 transmembrane domain also contains a GxxxG motif which is known to enable Glycophorin A to form disulfide-independent dimers [15]. It was therefore also possible, although far from certain [16], that this GxxxG motif contributes to SMIM1 dimer formation. Hence, in addition to using the cell-free system as an independent approach to investigate SMIM1 topology, we also exploited this technique to look at the molecular basis for the formation of SMIM1 dimers.

## Materials and methods

### Cloning

We cloned the SMIM1 wild-type (WT) cDNA into the pcDNA5/FRT/V5-His TOPO vector (Invitrogen, Carlsbad, CA, U.S.A.). For evaluation of N-glycosylation during *in vitro* translation we introduced at the N- or C-terminus of SMIM1 an 18 amino acid tag derived from bovine rhodopsin (OPG2) that contains two N-glycosylation sites (MNGTEGPNFYVPFSNKTG) [17,18]. Site-directed mutagenesis was done using *DpnI*-mediated mutagenesis [19]. SMIM1 WT does not have any N-glycosylation sites so novel sites were therefore introduced in the SMIM1 sequence by substituting either glutamine at position 4 or aspartic acid at position 15 for asparagine (Figure 1C). These positions were chosen since only a single amino acid change was required to introduce the consensus sequence for N-glycosylation, NXS/T. Both locations are also substantially further away from the transmembrane domain of SMIM1 than the minimum 12–14 amino acids shown to be needed for efficient N-glycosylation of integral membrane proteins [20]. Point mutations were also introduced at the sites predicted to be of importance for the observed tendency of Vel to run as a dimer protein under non-reducing conditions, specifically the cysteines at position 35, 43 and 77 to alanine, and the glycines in the transmembrane GxxxG motif to leucines. As control for N-glycosylation the tail-anchored protein Sec61β-OPG2 was used [21] while opsin-tagged version of the secretory protein Apelin (Apelin-OPG2) was used as a soluble secretory protein to confirm the effectiveness of treatment with alkaline sodium carbonate [18]. RNA was produced using T7 RNA Polymerase (Promega, Madison, WI, U.S.A.) according to the manufacturer’s instructions.

### *In vitro* translation experiments

*In vitro* protein synthesis was performed as previously described [12,18]. Briefly, rabbit reticulocyte lysate (Promega) was supplemented with 19 amino acid mix and [^35^S] methionine/cysteine in the presence of canine pancreatic microsomes (gift from Professor Bernhard Dobberstein, University of Heidelberg). The mix was incubated at 30°C for 15 min, aurintricarboxylic acid added to 0.1 mM to stop further translation initiation, and samples incubated for an additional 15 min. Membranes were isolated by centrifugation through a high salt cushion (750 mM sucrose, 500 mM KOAc, 5 mM Mg(OAc)_2_, 50 mM HEPES-KOH pH 7.9) at 100000×***g*** for 10 min. Pelleted microsomes were resuspended in 0.1 M Na_2_CO_3_, pH 11.5 and incubated for 15 min on ice to enrich for integral membrane proteins [22], the membrane fraction re-isolated by centrifugation and resuspended in Laemmli sample buffer. To assess protein N-glycosylation, microsomes were directly resuspended in Laemmli sample buffer and treated with Endoglycosidase H (EndoH, New England Biolabs, Ipswich, MA, U.S.A.) or buffer control at 37°C for 4 h. Proteins were separated using sodium dodecyl sulfate/polyacrylamide gel electrophoresis (SDS/PAGE), the gels were then fixed and dried then exposed on phosphor-imager plates which were read using a Fuji FLA-3000 Phosphor-Imager (Fujifilm, Tokyo, Japan).

### Cell culture and transfection

HEK293T/17 cells were cultured in Dulbecco’s modified Eagle’s medium with high glucose, l-glutamine and Phenol Red (Thermo Scientific, Waltham, MA, U.S.A.) supplemented with 10% fetal bovine serum (Thermo Scientific) at 37°C and 5% CO_2_. Cells at 60% confluence were transfected with SMIM1 variants in the pcDNA5/FRT/V5-His TOPO vector using the jetPRIME kit (Polyplus, Illkirch, France) and then cultured for 48 h before analysis.

### Western blot

Transfected cells were resuspended in 2× Laemmli buffer (Bio-Rad, Hercules, CA, U.S.A.) supplemented with 5% β-mercaptoethanol (β-ME). Cells were then lysed through sonication and either frozen at −80°C until enzyme treatment or heated at 95°C and briefly spun in a microfuge before the cell lysate was loaded on to a gel. We performed SDS/PAGE according to the Bio-Rad V3 system workflow using Bio-Rad products unless otherwise specified. Stain-free Tris-Glycine eXtended (TGX; any kDa) 10-well mini gels were used and resolved proteins were transferred to PVDF-membranes using the Trans-blot Turbo transfer system. The membranes were blocked in 5% non-fat milk in Tris-buffered saline with 0.1% Tween (TBS-T, Sigma–Aldrich, Saint Louis, MO, U.S.A.). The membranes were incubated with primary antibodies at 4°C overnight and secondary antibodies at room temperature for 1 h. We used ECL Clarity Western for developing the bands which were visualized in the ChemiDoc Touch Imager.

### Antibodies

For immunoblotting we used anti-SMIM1, a polyclonal antibody raised in rabbit against amino acids 1–15 in SMIM1 [2] diluted 1:25000 and HRP–conjugated goat-anti-rabbit IgG (Bio-Rad,) diluted 1:3000. All antibodies were diluted in 1% non-fat milk (Bio-Rad) in TBS-T. The anti-opsin tag antibody is a mouse monoclonal IgG antibody originally described in [23], now produced in-house and used at 1:1000 together with HRP–conjugated goat-anti-mouse IgG (Bio-Rad) (1:3000). Flow cytometry analysis of opsin was done with the same primary anti-opsin antibody diluted 1:100, but with APC–conjugated goat-anti-mouse IgG at 1:200 (Jackson ImmunoResearch, Cambridge, U.K.) and the Vel expression was analyzed with the human IgG monoclonal anti-Vel [5] at 1:100 with APC–conjugated goat-anti-human IgG (Jackson ImmunoResearch) at 1:200.

### Flow cytometry

Antibody staining of HEK293T/17 cells was done in 1% bovine serum albumin (BSA, Sigma–Aldrich) in TBS at room temperature for 10-min shaking. Cells were washed in phosphate-buffered saline (PBS) twice between staining and resuspended in 1% BSA/TBS before analyzed on the FACS CantoII (BD Scientific, Franklin Lakes, NJ, U.S.A.). The data were processed using FCS Express 6 (DeNovo Software, Pasadena, CA, U.S.A.).

### Enzymatic treatment of cell lysates

Glycosidase treatment was performed on cell lysates using either EndoH (Sigma–Aldrich) or Peptide:N-glycosidase F and Neuroaminidase (PNGaseF, Sigma–Aldrich) in the presence of 1% Triton-X100 (Sigma–Aldrich). Samples were denatured in sample buffer with 1% SDS and 5% β-ME. As reaction mixture, 80 mM mono- and disodium phosphate was used according to instructions of the manufacturer. Samples were heated at 95°C before the enzymes were added and reactions incubated at 30°C for 3 h. Laemmli sample buffer was added and proteins were separated on SDS/PAGE and blotted as described above.

## Results

### Cell-free expression of SMIM1

**Figure 2:**
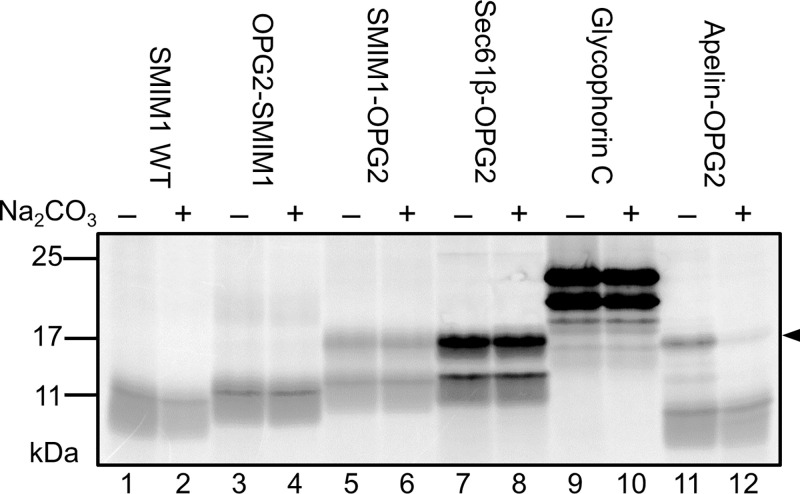
SMIM1 is an integral membrane protein SMIM1 was translated in a cell-free system in the presence of ER-derived microsomes, the membrane fraction was treated with sodium carbonate (Na_2_CO_3_), re-isolated and separated on SDS/PAGE. SMIM1 WT and its two opsin-tagged SMIM1 variants, as well as the known integral membrane proteins Sec61β and Glycophorin C are all strongly resistant to Na_2_CO_3_ treatment, consistent with them being integral membrane proteins. The N-glycosylated, ER luminal form of the soluble secretory protein Apelin (see arrowhead), is extracted from the membrane after treatment (lane 12).

First, we wanted to analyze the membrane insertion of SMIM1 to investigate whether it behaves as a *bona fide* transmembrane-spanning protein as opposed to a membrane-associated/monotopic protein [12] (Figure 1B). We therefore translated SMIM1 *in vitro* in the presence of ER-derived dog pancreatic microsomes and treated the resulting membrane-associated fraction with alkaline sodium carbonate solution in order to extract both peripheral/membrane-associated and fully translocated ER-luminal proteins while leaving integral membrane proteins largely untouched [22]. The SMIM1 WT protein, together with both versions of opsin-tagged SMIM1 (OPG2-SMIM1 and SMIM1-OPG2) behaved like the known integral membrane proteins, Sec61β-OPG2 and Glycophorin C, and remained in the membrane fraction after sodium carbonate treatment (Figure 2, lanes 1–10). In contrast, the N-glycosylated, membrane translocated, Apelin-OPG2 was almost completely removed from within the ER microsomes by the same treatment (Figure 2, lanes 11 and 12). On this basis, we conclude that SMIM1 behaves as a *bona fide* integral membrane protein and that inclusion of the OPG2 tag did not affect this behavior.

**Figure 3:**
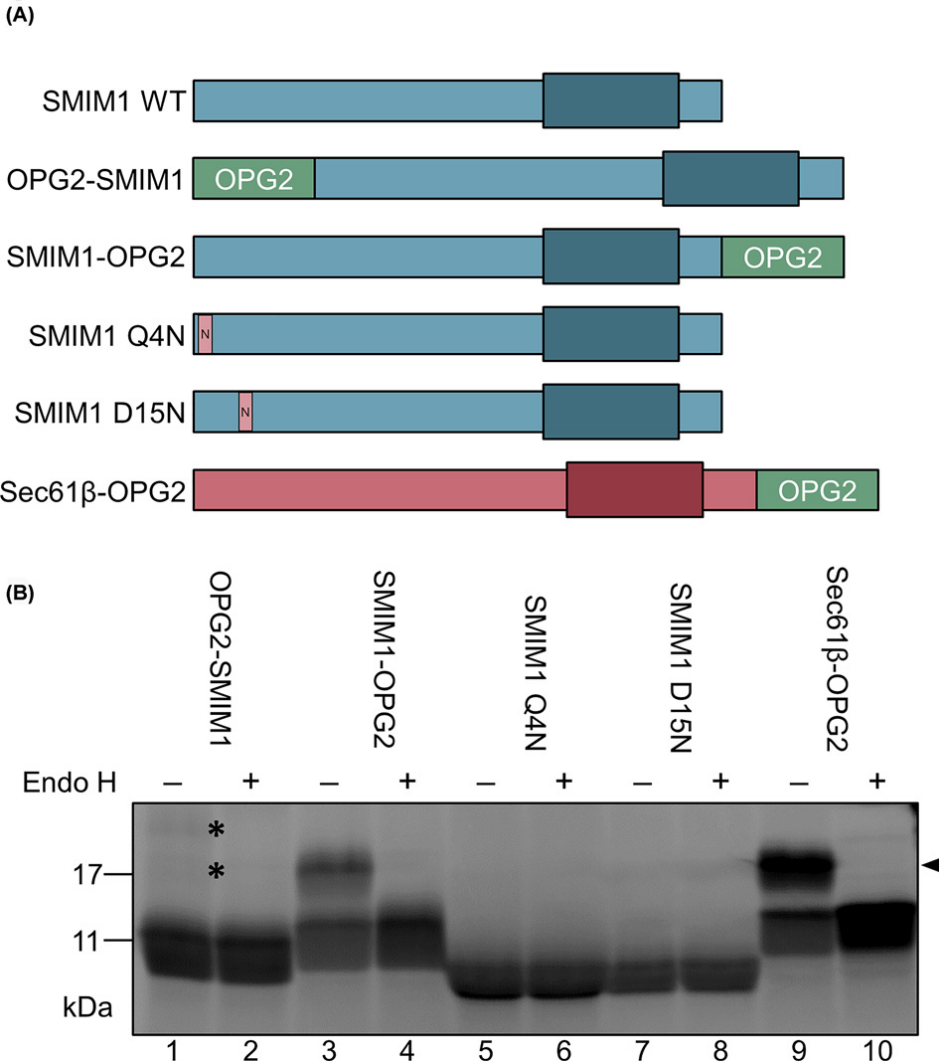
The majority of in vitro translated opsin-tagged SMIM1 is inserted with its C-terminus translocated into microsomes, but some are inserted with the N-terminus translocated (**A**) SMIM1 and Sec61β constructs with N-glycosylation sites either in the OPG2 tag or inserted as point mutations. Transmembrane region indicated as wider square. (**B**) SMIM1 constructs translated *in vitro* in the presence of microsomes. N-glycosylation in microsomes during translation is detected by change in mobility upon EndoH treatment. For SMIM1-OPG2, where the opsin tag is at the C-terminus, the majority of the membrane-associated protein is N-glycosylated (cf. lanes 3 and 4, see arrowhead). For the N-terminal opsin tag, two very faint EndoH sensitive products are detected (cf. lanes 1 and 2, see asterisks), indicating that a small fraction of OPG2-SMIM1 has inserted with its N-terminus in the lumen of the ER microsomes.

We next analyzed the N-glycosylation of SMIM1 variants (Figure 3A) by treating the radiolabeled proteins with EndoH in order to identify N-glycosylated products on the basis of their change in mobility following de-glycosylation. For the two SMIM1 constructs where we had created artificial N-glycosylation sites at residues 4 (Q4N) or 15 (D15N) in the N-terminal domain of the protein, no EndoH-sensitive N-glycosylated products were detected (Figure 3B, lanes 5–8). However, when the OPG2 tag was added to the N-terminus of SMIM1 (OPG2-SMIM1) two extremely faint, EndoH sensitive products were apparent (Figure 3B, lanes 1 and 2). In contrast, when the same OPG2 tag was added to the C-terminus of SMIM1 (SMIM1-OPG2), we observed robust levels of N-glycosylation, comparable with that seen when the OPG2 tag is added to the *bona fide* tail-anchored membrane protein Sec61β (Figure 3B, cf. lanes 3, 4, 9 and 10). On the basis of these *in vitro* data, we conclude that the majority of the SMIM1 protein is inserted into the ER membrane with the same orientation as the well-characterized tail-anchored protein Sec61β, and hence its N-terminus remains on the cytoplasmic side of the membrane. The low levels of N-glycosylation we observed when the OPG2-tag is located on the N-terminus of SMIM1 (OPG2-SMIM1) indicate that a small proportion of the protein may assume the opposite transmembrane topology with its C-terminus remaining cytoplasmic, when synthesized in a cell-free system, raising the possibility that it might display some degree of dual-topology (cf. Figure 1B, iv). In order to establish the physiological relevance of these *in vitro* findings in relation to the plasma-membrane resident SMIM1 found in erythroid cells, we next investigated the topology of our OPG2-tagged SMIM1 variants in the plasma membrane of mammalian cells.

### Opsin-tagged SMIM1 expressed in cell line HEK293T/17

**Figure 4:**
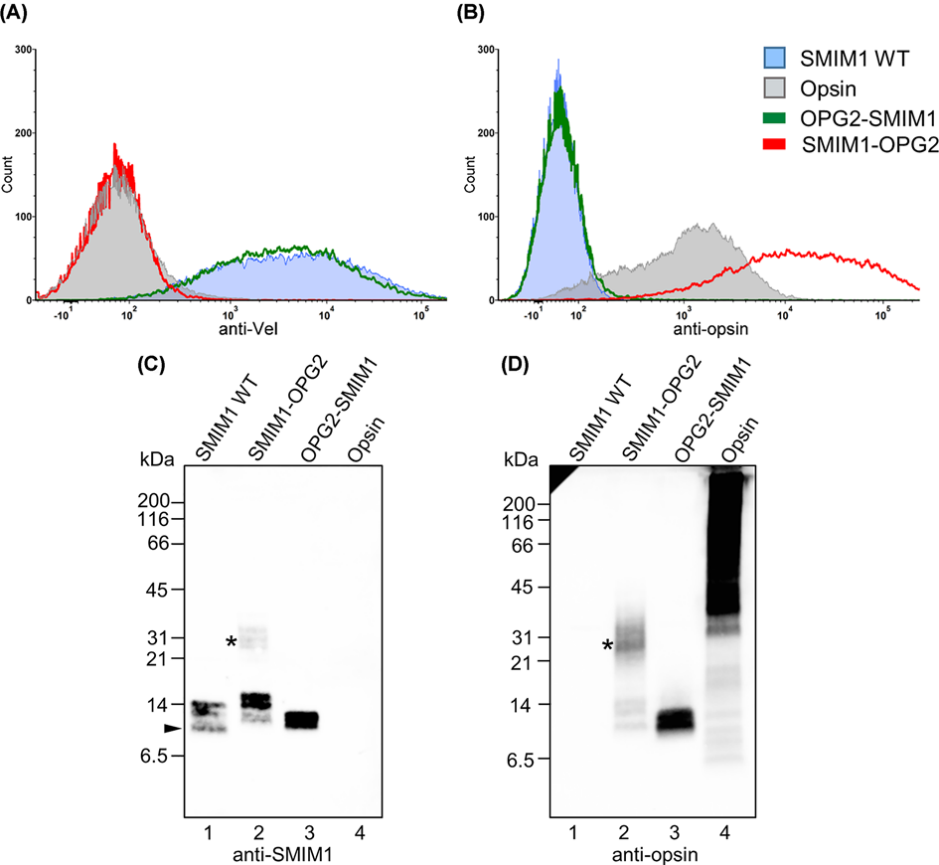
SMIM1 with opsin tag has a tail-anchored transmembrane orientation in cell line experiments SMIM1 with or without opsin tag transiently expressed in HEK293T/17 cells. Flow cytometry with anti-Vel (**A**) and anti-opsin (**B**). Only the OPG2-SMIM1 with the opsin tag at its N-terminus allows detection of Vel epitope (A). The opsin tag is only exposed on the cell surface when attached to the C-terminus of SMIM1-OPG2 (B). (**C,D**) Western blot after SDS/PAGE separation of lysed HEK293T/17 under reducing conditions with anti-SMIM1 (C) and anti-opsin (D). SMIM1 WT at ∼10 kDa with a smear composed of phosphorylated SMIM1 marked with arrowhead (C, lane 1). For SMIM1-OPG2 (C and D, lane 2) a heavier than expected band at ∼31 kDa is also present, marked with asterisks. Due to heating of the samples prior to SDS/PAGE, a proportion of the opsin control protein formed higher molecular weight aggregates (D, lane 4).

The transfected HEK293T/17 cells were harvested after 48 h and samples were analyzed by flow cytometry and Western blot. Cells transiently expressing WT bovine opsin labeled with anti-Vel, and cells transiently expressing SMIM1 WT labeled with anti-opsin were both confirmed as negative by flow cytometry, confirming the antibody specificity and the absence of any endogenous proteins bearing the relevant epitopes (Figure 4A,B, respectively).

Anti-Vel detected the Vel epitope on cells transfected with either the SMIM1 WT or OPG2-SMIM1 construct, but not on cells expressing SMIM1-OPG2 (Figure 4A), supporting the principal conclusion of our *in vitro* studies, namely that the extracellular Vel epitope resides in the short C-terminal domain of SMIM1 (cf. Figure 1B). Furthermore, our data suggest that the Vel epitope in the SMIM1-OPG2 construct becomes inaccessible for binding by the anti-Vel since no Vel is detected (Figure 4A), most likely due to the C-terminal location of the OPG2 tag (cf. Figure 3A).

Although the OPG2 tag was readily detectable in its endogenous location of the N-terminal extracellular domain of opsin (Figure 4B), when the OPG2 tag was appended to SMIM1 it could only be detected on the cell surface when it was at the C-terminal end of the protein, i.e. in SMIM1-OPG2, (Figure 4B). Hence, our flow cytometry analysis directly supports our *in vitro* data indicating that SMIM1 is a tail-anchored protein with its short C-terminus exposed outside the plasma membrane. Using our cell-based system we were unable to detect any SMIM1 molecules with the opposite orientation (N-terminus extracellular) as judged by the availability of the OPG2 tag for antibody binding to OPG2-SMIM1. This suggests that SMIM1 does not assume a dual topology at the plasma membrane.

In order to confirm the presence of the relevant SMIM1 variants analyzed by flow cytometry we carried out immunoblotting studies. When an antibody directed toward the 15 most N-terminal amino acids of SMIM1 was used, it detected SMIM1 WT products of the expected MW ∼10 kDa (Figure 4C, lane 1) with a smear between ∼10 and 14 kDa corresponding to the phosphorylated species of SMIM1 [7]. SMIM1 WT was not recognized by the anti-OPG2 specific monoclonal antibody (Figure 4D, lane 1). Irrespective of which end of SMIM1 the OPG2 tag was attached to, immunoblotting confirmed that both the anti-SMIM1 and anti-opsin antibodies detected products with a slightly higher molecular weight than the smallest of the products seen with SMIM1 WT, likely reflecting the addition of the OPG2 epitope tag (Figure 4C,D, lanes 2 and 3). An additional product of ∼31 kDa observed following expression of SMIM1-OPG2, and detected with both antibodies (Figure 4C,D, lane 2), was explored further to clarify its nature.

### Investigation of post-translational modifications on opsin-tagged SMIM1

**Figure 5:**
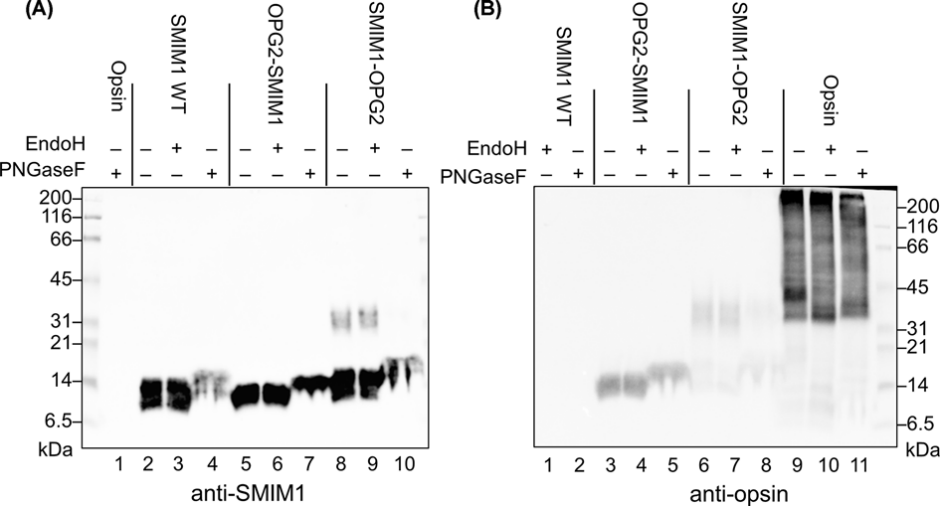
SMIM1 with C-terminal opsin tag is modified with a glycan sensitive to PNGaseF treatment SMIM1 with and without opsin tag, as well as opsin control, expressed in HEK293T/17 cells. Cell lysates were treated with EndoH or PNGaseF then separated on SDS/PAGE and blotted with anti-SMIM1 (**A**) or anti-opsin (**B**). No N-glycosylation or glycosidase effect is seen with regard to OPG2-SMIM1 (A, lanes 5–7 and B, lanes 3–5). However, SMIM1-OPG2 with the tag at the C-terminus is affected by PNGaseF, where the band disappears in blots with anti-SMIM1 (A, lane 10) and anti-opsin (B, lane 8). The de-glycosylation of the N-glycosylated opsin control by both glycosidases (B, lanes 10 and 11) can be seen despite the presence of some higher molecular weight aggregates. The general perturbation of SMIM1 migration following PNGaseF treatment is most likely due to the high amounts of Triton X-100 that need to be used during the de-glycosylation reaction.

We hypothesized the band at ∼31 kDa for SMIM1-OPG2 may correspond to its N-glycosylation. To confirm the orientation of plasma membrane-inserted SMIM1 variants and to characterize any potential post-translational modifications that occurred in HEK293T/17 cells, we used differential deglycosylation. Hence, samples were treated with two N-glycosidases: first, EndoH known to cleave high-mannose and hybrid glycans but leave the complex glycans untouched; second, PNGaseF that cleaves both high-mannose/hybrid glycans and N-linked glycans that have been further processed during transport through the Golgi complex en route to the plasma membrane. EndoH treatment had no effect on the mobility of SMIM1 WT, OPG2-SMIM1 or SMIM1-OPG2 products following detection with either anti-SMIM1 and anti-opsin (Figure 5A, lanes 2–10 and Figure 5B, lanes 1–8). However, PNGaseF treatment strongly diminished the ∼31 kDa product observed with SMIM1-OPG2 (Figure 5A, lanes 8–10 and Figure 5B, lanes 6–8), confirming that this corresponds to the complex N-glycosylation of the C-terminal tag present in the SMIM1-OPG2. In combination with our ability to detect the OPG2-tag of SMIM1-OPG2 at the plasma-membrane by flow cytometry (Figure 4B) we conclude that this opsin-tagged SMIM1 construct is trafficked from the ER through the Golgi and onward to the plasma membrane during expression in HEK293T/17. Interestingly, following expression in HEK293T/17 cells we find that the mobility of bovine opsin is sensitive to treatment with both EndoH and PNGaseF (Figure 5A, lane 1 and Figure 5B, lanes 9–11). Since we can detect opsin in the plasma membrane (cf. Figure 4B), our data suggest that when expressed in this heterologous system, any opsin that reaches the cell surface retains high-mannose and/or hybrid type N-linked glycans.

### *In vitro* translated SMIM1 forms homodimers under non-reducing conditions

**Figure 6:**
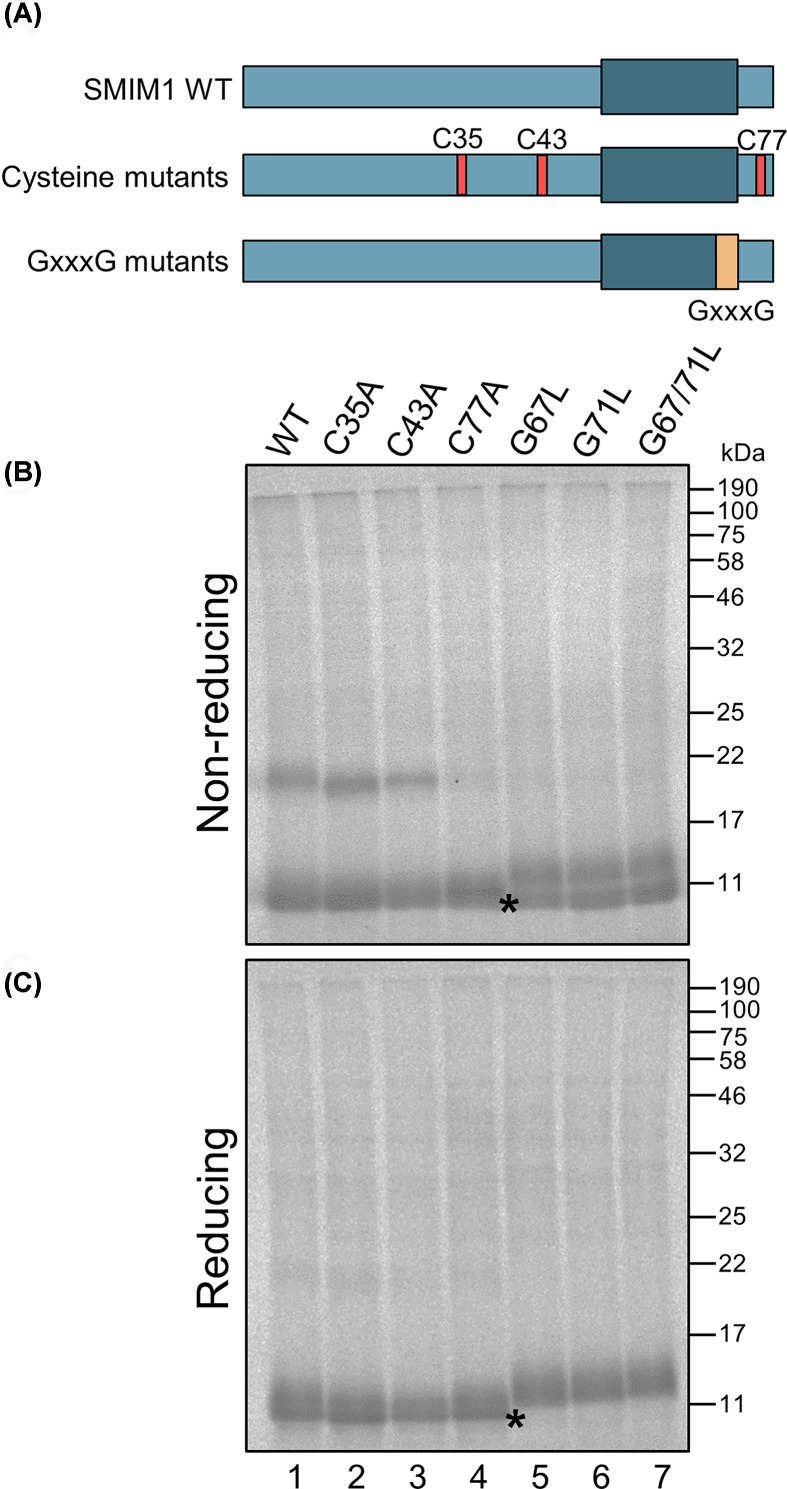
SMIM1 translated *in vitro* forms homodimers sensitive to point mutations (**A**) SMIM1 WT constructs with point mutations at either of the cysteines 35, 43 and 77 substituted for alanine, and the GxxxG motif in the transmembrane region with one or both glycines substituted for leucines. (**B,C**) SMIM1 constructs translated in the cell-free system and separated on SDS/PAGE with non-reducing (B) or reducing (C) Laemmli sample buffer, were detected by phosphorimaging. (B) The homodimer band (20 kDa) under non-reducing conditions cannot be seen when the cysteine at position 77 is mutated but is unaffected by mutations of the other cysteines. The mutated glycines in the GxxxG motif all inhibit dimerization. A faster migrating species of the G to L mutants present only under non-reducing conditions (B) and absent under reduced conditions (C) is indicated by asterisks and may reflect the formation of an intrachain disulfide bond. Similarly, the presumptive dimers are also no longer present (C).

SMIM1 was efficiently expressed in the *in vitro* translation system and typically generated broad bands that often resolved into two closely spaced products under normal reducing conditions (e.g. Figure 3B, lanes 1–8). We noted that when the same products were run under non-reducing conditions we could detect substantial levels of a higher molecular weight species that migrated as a potential dimer of SMIM1 (Figure 6B,C, lane 1). Given that this dimer was only seen under non-reducing conditions we concluded that it was most likely stabilized by a disulfide bond between two SMIM1 monomers and therefore set out to test this hypothesis via mutagenesis (Figure 6A). To this end, cysteine residues 35, 43 and 77 were individually mutated to alanine and the effect on SMIM1 dimerization determined. Our data strongly suggest that Cys^77^ is responsible for the disulfide-bonded SMIM1 dimer that we detect *in vitro*, since the dimer is completely absent from this SMIM1 mutant (Figure 6B, lane 4). We noted two glycine residues that were located adjacent to Cys^77^ which appeared to conform to the GxxxG motif previously implicated in the dimerization of Glycophorin A [15]. Mutation of glycines 67, 71 or both, to a bulkier leucine residue completely abolished the formation of SMIM1 dimers even when Cys^77^ was present (Figure 6B, lanes 5–7). Interestingly, when the glycine mutants of SMIM1 were resolved under non-reducing conditions, a doublet of products that resolved into a single species under reducing conditions was seen. We speculate that an additional, apparently faster migrating species observed under non-reducing conditions (Figure 6B, lanes 5–7), may represent SMIM1 proteins with a non-native intrachain disulfide bond, perhaps formed as a consequence of their inability to form an authentic SMIM1 dimer. This possibility will require additional experimental investigation beyond the scope of our current study. Nevertheless, we provide evidence that SMIM1 can assemble into a disulfide-bonded dimer during its biogenesis at the ER.

## Discussion and conclusions

The genetic background of the Vel blood group was simultaneously elucidated in 2013 by three different research groups [2–4]. The *SMIM1* gene, previously known by the name *LOC388588* was thereby annotated in the human genome, which opened up the way for the genetic correlation between the 17-bp deletion in *SMIM1* and the Vel– phenotype. Subsequent studies have focused on genetic regulatory regions of the gene and its protein expression [24,25], genetic screening approaches in different populations to identify more Vel– blood donors [11] together with the biochemical properties of SMIM1 [7]. The small size of SMIM1, with a very short extracellular C-terminal domain, initially seemed unlikely to be the sole explanation for this highly immunogenic protein that resides at the RBC surface. Such a topology also made it difficult to speculate on the mechanisms behind any of the suggested functions of SMIM1, since they appeared to require a more substantial extracellular domain than a tail-anchored transmembrane protein could provide.

Taken together with the conflicting computational predictions for the membrane orientation of SMIM1 we considered it important to investigate the topology of SMIM1 using an *in vitro* expression system that would complement previous cell-based studies [7] and allow us to address a range of possible interactions between SMIM1 and the plasma membrane (Figure 1B). The *in vitro* expression of SMIM1 with opsin tags to allow for N-glycosylation showed a clear preference for SMIM1 to orient in cellular membranes with its N-terminus located in the cytosol, resulting in its C-terminus being extracellular following the trafficking of newly synthesized SMIM1 to the plasma-membrane. Although we could detect a small fraction of SMIM1 with the opposite orientation *in vitro*, our subsequent cell-based studies using HEK293T/17 showed that the only SMIM1 detectable in the plasma membrane is oriented with its C-terminus exposed to the outside of the cell. The modest indications of dual-topology for SMIM1 that we observed in the cell-free expression system may reflect a small reduction in the fidelity of membrane insertion or result from the addition of the C-terminal OPG2 tag that we used as a reporter for translocation into the ER lumen [26]. Alternatively, it may be that a small population of SMIM1 assumes an alternative topology when expressed in cells, but the resident ER quality control system recognizes this form as erroneous and elicits its selective degradation [27]. Likewise, any SMIM1 constructs in our cell-based analysis that appear distinct from the native protein would be caught in the ER protein control pathway as misfolded or non-native, and retained in the ER for later degradation via the ubiquitin–proteasome system [28]. However, our flow cytometry studies show that both of our OPG2-tagged SMIM1 variants are efficiently expressed at the plasma membrane (Figure 4A,B), and on this basis we conclude that they behave like the native SMIM1 protein. Hence, robust conclusions regarding the membrane orientation of SMIM1 can be made by studying these variants.

Both our *in vitro* and cell-based studies are in close agreement with a previous study of SMIM1 topology [7] and suggest that at the erythrocyte plasma membrane the C-terminus of SMIM1 is exposed to the outside of the cell. Given the small number of amino acids between its transmembrane domain and C-terminus (Figure 1C) it seems likely that SMIM1 is actually a tail-anchored protein and it may therefore normally be inserted post-translationally into the ER membrane [26]. The results of our glycosidase treatments indicate that following membrane insertion at the ER, SMIM1 is trafficked through the Golgi complex *en route* to the plasma membrane. Since the majority of the SMIM1 protein, in the form of its N-terminal region, resides inside the cell, we speculate that this domain may contribute to the function of SMIM1, as is typically the case for other tail-anchored proteins, for instance reminiscent of the SNARE proteins which mediate membrane fusion during intracellular membrane trafficking [29]. Furthermore, our flow cytometry results confirm that the epitope for the Vel blood group antigen resides on the C-terminus of the SMIM1 protein, since when the opsin tag is attached to its C-terminus, the Vel epitope can no longer be detected, despite the efficient membrane insertion and cell surface expression of the tagged protein as judged using the opsin antibody. We therefore conclude that the opsin tag blocks the antigenic epitope, thereby inhibiting the binding of the anti-Vel antibody.

At last, we present *in vitro* expression data showing that Cys^77^ together with glycines 67 and 71 are important for the formation of disulfide-bonded SMIM1 homodimers that can be observed under non-reducing conditions. This behavior further supports our conclusion that SMIM1 is a tail-anchored protein since the formation of disulfide bridges is catalyzed by oxidoreductases located in the ER lumen [30]. If the C-terminus of SMIM1 is indeed translocated into the ER lumen as our data show, then the only cysteine available for catalyzed disulfide bond formation inside the ER lumen is Cys^77^ (cf. Figure 1C). Based on predictions of the transmembrane domain of SMIM1, the GxxxG motif resides inside the membrane. This motif has been well described in the erythroid transmembrane protein Glycophorin A [15], and its ability to facilitate dimer formation can be influenced by both sequence context, i.e. the other amino acids located in proximity, and the lipid bilayer [31]. In the case of the lipid bilayer it has been argued that it can be seen as the ‘solvent’ in which the transmembrane domain dimers form. In the case of Glycophorin A, although this membrane environment will have an effect, it is the Van der Waal’s forces and hydrogen bonds between amino acid side chains that are the key drivers of the interaction [31]. In addition, evaluation of the GxxxG motifs in different proteins has shown that they can also take part in dimerization of soluble proteins as well as proteins in detergent micelles, further supporting the idea that the transmembrane domains contribute to, but is not essential for, dimer formation through the GxxxG motif [16]. The dimerization of SMIM1 through Cys^77^ and the GxxxG motif is evident from our *in vitro* studies, and this behavior has potential physiological significance, since the Vel blood group antigen is also prone to dimer formation under non-reducing conditions [2].

In short, our *in vitro* study of SMIM1 biogenesis and membrane topology is the first to employ a cell-free system, from which we can conclude that SMIM1 has a tail-anchored protein topology established during synthesis at the ER and maintained during transport to the plasma membrane. We speculate that the larger N-terminal domain located in the cytosol may contribute to the as yet unknown function(s) of SMIM1. Our results from this complementary approach agree well with a previous study of SMIM1 topology using cell-based expression [7]. Our studies also show that the formation of SMIM1 dimers occurs at the ER and that the dimer is stabilized by a native disulfide bridge formed between the short C-terminal domains of two SMIM1 polypeptides. This most likely reflect the *bona fide* pathway for the biosynthesis of the Vel blood group antigen that our recent parallel studies have revealed [32].

## Abbreviations

APC: allophycocyanin
Apelin-OPG2: opsin-tagged version of the secretory protein Apelin
BSA: bovine serum albumin
EndoH: endoglycosidase H
ER: endoplasmic reticulum
HRP: horseradish peroxidase
OPG2: opsin-derived 18 amino acid tag with N-glycosylation sites
PNGaseF: peptide:N-glycosidase F
RBC: red blood cell
SDS/PAGE: sodium dodecyl sulfate/polyacrylamide gel electrophoresis
SMIM1: small Integral Membrane Protein 1
TBS-T: tris-buffered saline with 0.1% Tween
Vel−: vel negative
WT: wild-type
β-ME: β-mercaptoethanol
